# Queens and Wet Nurses: Indispensable Women in the Dynasty of the Sun King (1540–1580)

**DOI:** 10.3390/healthcare10020316

**Published:** 2022-02-07

**Authors:** Sagrario Gómez-Cantarino, Laura Romera-Álvarez, Mercedes de Dios-Aguado, María Idoia Ugarte-Gurrutxaga, José Siles-Gonzalez, Maylene Cotto-Andino

**Affiliations:** 1 Faculty of Physiotherapy and Nursing, Toledo Campus, University of Castilla-La Mancha, 45071 Toledo, Spain; sagrario.gomez@uclm.es (S.G.-C.); lauraromera140697@gmail.com (L.R.-Á.); 2 ENDOCU Research Group (Nursing, Pain and Care), Department of Nursing, Physiotherapy and Occupational Therapy University of Castilla-La Mancha, 45071 Toledo, Spain; 3 Health Sciences Research Unit: Nursing (UICISA: E), Coimbra School of Nursing (ESEnfC), 3004-011 Coimbra, Portugal; mded@sescam.jccm.es (M.d.D.-A.); PROFESOR.MCotto@uclm.es (M.C.-A.); 4 Sillería Health Center, Health Service of Castilla-La Mancha, Av. Santa Reliquia 26, 45313 Toledo, Spain; 5 Department of Nursing, University of Alicante, Carretera de San Vicente del Raspeig s/n, 03690 Alicante, Spain; jose.siles@ua.es; 6 Language Immersion and Promotion, Language Centre, Toledo Campus, University of Castilla-La Mancha, 45071 Toledo, Spain

**Keywords:** special care, woman, mother, health promotion, breastfeeding, historical research, wet nurse, society, maternal, infant

## Abstract

In Spain, the wet nurse had a prominent place in the Court of Philip II (1540–1580), suckling princes. The aim of this review is to identify the role of wet nurses in the Spanish monarchy and the survival of the infants, who were children of Philip II (16th century). A scoping review is presented, studying documents on wet nurses in the Spanish monarchy. The dialectical structural model of care (DSMC) is applied, and three thematic blocks are used to make up the historical-cultural model. Books, chapters and databases were analysed from Cuiden, Pubmed, Scopus, Science Direct and Google Scholar, from January–September 2021. These wet nurses were treated as ladies, as they came from wealthy families related to royalty. The services of wet nurses from neighbouring localities to the court were used. They had to be of good appearance and in excellent health. They were hired because of the need for survival of the infants, children of Philip II. The functions of the four wives of Philip II were relegated to reproduction, childcare, family and monarchical duties. They used empirical medicine in the form of prescriptions for beauty, hygiene and feminine care. The wet nurses were the driving force that promoted the health of babies through breastfeeding.

## 1. Introduction

The wet nurse’s job is possibly one of the oldest jobs performed by women, and yet one of the least known. This type of work was known as mercenary nursing, which consisted of the economic remuneration of a woman in exchange for breastfeeding a child of another, for the survival of the family [1,2]. The etymological origin of wet nurse comes from nutricia, the economic remuneration that these women received for their work [2]. To study the figure of the wet nurse, her representation and her role in history, a retrospective study is needed to uncover the origin of the concept and the relevant contribution of wet nurses to the generations since, starting from almost the beginning of humanity. This historical research article describes the role of the wet nurse in Europe, particularly in Spain, among royal families. Specifically, it studies this figure during the period 1540–1580, during the reign of Philip II. Their legacy was inherited from Greco-Latin, Christian and, especially, Arab culture. There is evidence of their presence in the Ebers Papyrus (1550 BC) and in Babylonian codes, such as those of Hammurabi and the Laws of Esnunna [3,4,5]. Thus, this trade was socially integrated in Ancient Greece and Rome, where it was the wet nurse who managed her contract in the markets, specifically in the lactaria [6]. When looking at the figure of the wet nurse, the first thing to analyse is the term itself, which comes from the Greek “Τιθηνη’” and “τροφος’”, which are the words used to designate the Roman nutrix [7]. The word nutrix comes from the word nutricium, which was the office of a wet nurse, the etymological origin of which was nutricia, referring to the salary she received for her work. The term “τ ‘ιτθη”, or mistress of milk as it was properly called, differs from “τροφος’”, which designates what has been called the “dry mistress”. The term wet nurse had two meanings: one was to suckle other people’s children, and the other was to help in the upbringing of the newborn. This term, its meaning and the relevance of its figure already appear in the Homeric poems, especially in Homer’s Iliad [8]. The wet nurse of the Homeric era is an illustrious and cultured wet nurse, as indicated by the importance that the aedo gives to the nobility of his lineage. In the Iliad, for example, Euriclea, the wet nurse of Odysseus, is the prototype of the educated and celebrated wet nurse. She played a transcendental role throughout Ulysses’ life and in the poem.

In Ancient Rome, nutrix, wet nurses or nursemaids of children who were not their own, were the women who nourished children in their early infancy, cared for them and educated them as they grew up. The use of wet nurses became widespread in the late Republican period, when matrons of aristocratic rank acquired the custom of using the services of these women to nurse their children. The use of wet nurses became, during the Empire, a common practice [9].

Emphasising the relevance of these women, we can refer to Bartholomew the Englishman, who defined this figure in the 13th century as follows: “the mistress is called in Latin nutrix which means nurturer who brings up because she brought up the child from her milk, instead of his mother. For, just as if she were his mother, she heals him and aches for his pain and takes pleasure in his joy and she heals him” [10]. The “criadoras”, or wet nurses, were present in social institutions, homes and in class homes of all social classes. It is worth noting that the most privileged wet nurses were sought after by the aristocracy, becoming part of the families. This figure acquired an important role in the Spanish monarchy [11,12,13]. The requirements for working as a wet nurse were also established by the Church, which, following its precepts, demanded that they be healthy, honest and honest with their families, as well as being pleasant to the infant [13,14]. Such was the relevance of this profession in society that it was already regulated by law in the 12th century, specifically in the Royal Charter of Castile (1252–1255), where the rights of children were protected, thus increasing their survival (Siles, 1996). Despite this, infant mortality in Spain in the 16th century was very high, due to reasons that included famine caused by poor harvests, years of drought and inflation in the prices of essential products, as a result of food imported from abroad [15]. This situation was more marked in certain geographical areas of Spain, with a death rate of 45% for infants and young people, known from sacramental books through ecclesiastical registers, which at the time reported the natural movements of the population [16]. It is true that in the upper social classes, infant mortality was due to endemic diseases of the area, such as infectious, digestive and pulmonary diseases, among others [17,18]. A clear example of this is the deaths of four children (three boys and one girl) of Queen Anne of Austria, the fourth wife of Philip II [19]. Therefore, the figure of the wet nurse promoted the survival of the infants, as the queens had to have children in a short period of time, thus ensuring the continuity of the crown, a situation that not only weakened them, but also made it impossible for them to breastfeed [20]. There was even a belief that the milk would be contaminated by sexual intercourse, which contraindicated marital relations during the period of lactation, a situation that the queens could not afford. Given these extreme circumstances for the queens, the responsibility for choosing the wet nurse fell on the queen herself, in addition to the queen’s mother, a figure who was sometimes of vital importance in advising the queen on intimate sexual and health care matters [19]. The economic remuneration received by these women was substantial, as the average salary amounted to 272 maravedíes per month, a high amount in comparison with other female occupations of the time, as the average salary of a servant was 700 maravedíes per year [1]. These women also received as payment for their services the bed used by them, as well as the cradle and even the infant’s bed. They also received clothing, which consisted of dresses, shirts and bed linen [21].

In general terms, these women were treated as ladies, as most of them came from wealthy families who were related to royalty. On the other hand, the services of wet nurses who lived in towns adjacent to Madrid, such as Toledo, Illescas, Valdemoro, Torrejon and Segovia, were also used. Some of these women even breastfed several infants, considering themselves “milk siblings”, both among themselves and with their children. Thus, the relevance of this profession in the high society of the years 1540–1580 is demonstrated [19,20,21].

Further into the modern and contemporary era, the wet nurse was also known as the “milkmaid”, whose sole function was to breastfeed the newborn every three to four hours. She was also known as a “nursemaid” when performing other types of occupations, such as education, entertainment and care; nursemaids received a generally defined salary per month, which decreased when the child was fed by mixed breastfeeding [22]. The working hours of these women ranged from day only to night only to full-time care. There are even records of wet nurses who performed their duties in a completely altruistic manner, in the vast majority of cases solely out of love for the newborn or attachment to their family [23]. The time period studied in this research addresses the importance of offspring and newborn survival in the Spanish monarchy, in the period 1540–1580, where infant mortality, even among royal infants, was high, as was the need for a male heir to the throne in a Spain with great power (known as “the Empire where the sun did not set”). Medical knowledge, as well as the lack of social and health resources in that period (1540–1580), together with infectious and contagious diseases, made it very difficult for infants to survive. For this reason, the figure of the wet nurse guaranteed the good nutrition of the royal newborns, while allowing the queens to have frequent pregnancies, as they did not have to breastfeed their children. All these reasons made wet nurses a key profession, recognised on a social level and with greater impact in high society, which is why it is of historical interest to deal with these women in depth. 

In the following section, the methodology used to carry out the analysis in accordance with the research objectives is presented. The main objective is to relate the figure of the wet nurse within Spanish royalty with the survival of infants during the period from 1540 to 1580, coinciding with the reign of Philip II (known as the Sun King). The secondary objectives are (1) to identify the figure of the queen as a woman and mother; (2) to describe the figure of the royal wet nurse, her relationship with the infants and the benefits and retributions she acquired; (3) to classify natural remedies according to knowledge and beliefs in care. This is followed by a discussion, where the results are compared with previous knowledge. Finally, the main conclusions and future lines of research are discussed.

## 2. Materials and Methods

### 2.1. Study Design

**Figure 1:**
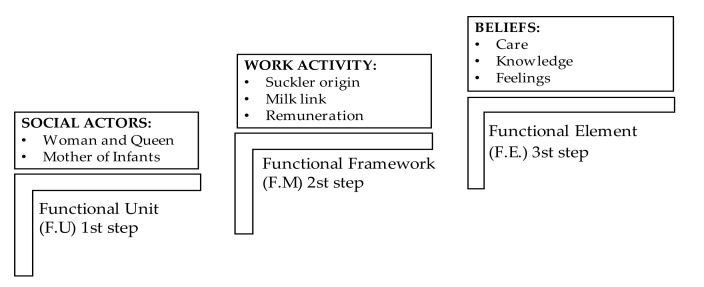
Theoretical dialectical structural model of care (DSMC) model: application of structures. Source: authors’ own elaboration.

In this article, a scoping review was conducted as a method to address the objective of the study. These reviews are an ideal tool for determining the scope of a set of publications on a given topic. They give an idea of the volume of publications and studies available. In addition to gaining insight into the focus of the papers, they allow researchers to evaluate, synthesise and critique the evidence inherent in the study target [24,25]. The Dialectical Structural Model of Care (DSMC) [26] was used for its relevance in delving into the cultural and social roots of the structures linked to the sexual and gender division of labour. This model identifies the functional dynamics of structures, allowing for the analysis of the causes that provoke their changes. The DSMC methodology is based on structures that support the process of organising and analysing the data. In this research, its application is important due to the social, cultural and care study involved. Therefore, the structures that are applied are (1) Functional Unit (FU), which in this case includes the social actors, both the woman and queen, the mother of infants; (2) Functional Framework (FF), related to the place of origin of the wet nurse and the link with the newborn, where the physiological and the labour aspects merge; (3) Functional Element (FE), where the care, knowledge and feelings that give rise to the social systems and that determine the sexual and gender division are included. These constitute a suitable tool for the organisation and analysis of the data, since the aim is to obtain concrete visions of a particular historical phenomenon from a cultural history perspective centred on the Spanish monarchy [27]. Within this research, three thematic blocks were developed, centred on the DSMC, with each of them encompassed within the structures that make up this historical and cultural model [28] (Figure 1).

### 2.2. Search Strategy

The process of a scoping review starts with an exploratory research question [29], aimed in this case at synthesising and systematically critiquing existing knowledge [30]. This review involved several steps [31]. Initially, the topic was identified and a research question was established, which was posed within cultural history and in the culture of the cui-dados by applying the Dialectical Structural Model of Care (DSMC): “what was the link between wet nurses during the reign of Philip II, specifically between 1540 and 1580?”. The inclusion criteria took the following into account: (1) publications in Spanish, English, Portuguese and Arabic; (2) there was no restriction on the time of publication, as this was historical research; (3) the subject matter was related to the figure of the wet nurse, the women of Philip II (1540–1580) and the empirical care of the period. The following were considered as exclusion criteria: (1) repeated publications; (2) abstracts, editorials, dissertations, course conclusion works and, (3) studies that did not address the proposed topic. 

Subsequently, a document search was carried out from January to September 2021. Libraries of the University of Castilla-La Mancha, the National Library of Spain, the Library of Castilla-La Mancha (Alcázar de Toledo), the National Historical Archive, the CSIC Library and the World Digital Library (UNESCO) were consulted. On the one hand, several sources were consulted: (1) Latin American Health Bibliographic Database (CUIDEN); (2) PubMed; (3) Scopus; (4) Science Direct; and (5) Google Scholar. MeSH and DeSH terms were used in order to carry out a more exhaustive and advanced search using the Boolean operator “[Y]”, “[AND]”, “[OR]”. In addition, word combinations were used where appropriate to reflect the syntax and search rules common to the individual databases. The descriptors were as follows: infant care, culture care, health promotion, breastfeeding, historical research, wet nurse, care, society, infant mortality and child survival. After searching, eliminating and selecting the articles, the selected articles were coded and identified (Table 1).

This scoping review was conducted from January to September 2021. This review aims to compile the evidence supporting a particular area of research and identify new documentation to help understand the topic of study. This type of review is not intended to analyse the methodological quality of the research included, nor to find the best scientific evidence. Rather, the aim is to thoroughly review the existing scientific evidence. Through the consensus of the authors (S.G.-C., L.R.-A., M.D.A., M.I.U.-G., J.S.-G., and M.C.A.), articles, books, proceedings, historical archives and chapters, among others, were reviewed. A total of 34 articles, 21 books, 7 book chapters and 3 conference proceedings comprised the documents that fulfilled the requirements reflected in the inclusion and exclusion criteria. A total of 64 documents relevant to the research were collected, as they covered the perspective of the three thematic blocks: queens, wet nurses and health care. In the end, a total of 66 documents fulfilling the requirements reflected in the inclusion and exclusion criteria were included.

### 2.3. Data Analysis

The content analysis of the documentation was carried out from a qualitative perspective through an objective and systematic critical reading of both manifest and latent content [28]. The steps carried out for the analysis consisted of (1) thematic linkage; (2) preliminary classification of the documents based on content and organisational criteria; (3) selection and extraction of relevant information according to the criteria of scoping reviews, with the purpose of enabling results and conclusions [28,29,30]. The selected articles were analysed from the point of view of the three thematic blocks studied, with each of them encompassed within the structures that make up the DSMC: (1) queen and need for offspring; (2) wet nurses and children of milk and fees received and (3) natural remedies: knowledge and beliefs in health. All these blocks are contextualised within the social reality prevalent in Spain during the period under study (1540–1580).

In order to extract and summarise the data in this historical research, an inferential interpretation was carried out by the researchers. An attempt was made to know the previously researched and written reality of the socio-health environment of the period studied in Spain. The first and second authors (S.G.C. and L.R.-A.) carried out the general extraction of the data. The third author (M.D.-A.) examined the findings. The fourth, fifth and sixth authors (M.I.U.-G., J.S.-G. and M.C.-A.) identified the common thematic lines, which are included within the structures that make up the DSMC, including its core (1st step) (UF), middle zone (2nd step) (MF) and outer part (3rd step) (EF). Where there was discrepancy in the choice and inclusion of research, this was resolved by consensus among the investigators. We did not exclude studies chosen for analysis on the basis of their degree of rigour, as the aim of the scoping review was to synthesise the results of the research reviewed in order to extrapolate greater knowledge and insight into health care to the scientific world [28]. Thus, after reading and re-reading the chosen articles, it was possible to answer the guiding pre-questions proposed in this research: “what was the degree of importance of the figure of the woman and queen linked to reproduction?” and “what was the link between the wet nurse and the children of Philip II?

### 2.4. Ethical Issues

The potential risks of this research are related to possible material damage to the documents consulted. Therefore, universal standards of care were followed in order not to damage the sources, for which personal protective equipment (gloves, glasses, masks, capes) were used when consulting the documents.

The documents consulted in the research were handled with care, caution and responsibility. The rules of the visiting libraries for the use and photocopying of their collections were also respected.

## 3. Results

The women and queens during the monarchy of Philip II (1540–1580) were supposedly healthy, of childbearing age and belonging to the European nobility, with the aim of reproduction, but with a particular meaning, because it was necessary and even obligatory to have male offspring in order to consolidate the reign and the ruling dynasty of the time. Therefore, these queens were carefully chosen, a situation that made it necessary to assess their origin in order to consolidate international relations for socio-political purposes, without forgetting the reproductive purpose [54]. They were all young healthy women from noble Catholic families, and most of them were related to the monarch. Thus, once the arranged marriage had taken place, they generally moved to the court, which was initially located in Toledo and later in Madrid, a situation that occurred from the third wife of Philip II onwards [20]. The functions of these queens were relegated to a life based on the provision of offspring as well as the care of children, the family and monarchical duties in the austerity of the time, a matter motivated by the continuous socio-political conflicts. This situation was not experienced by Isabella of Valois, the third wife of Philip II, a woman known for wearing the finest clothes due to her addiction to Franco-Spanish fashion. This woman even had taste for horse-riding and other sports, considered at the time to be exclusive to men [55].

Such was the importance of fathering a male in the Spanish monarchy of the time, to ensure the descendants of the crown, that some of these women were even buried in places other than the king, as a result of no giving him a male child. Such is the case of Queen Isabella of Valois, who requested to be buried in the Descalzas Reales monastery, a different place than her husband [54].

### 3.1. Woman and Queen: The Importance of Her Offspring

This section attempts to make visible and identify the figure of the queen as a woman and mother. Thus, this section deals with the wives of the monarch Philip II (1540–1580), with whom they had offspring in order to ensure a male heir to the throne to continue the dynasty. Philip II married for the first time to Maria Manuela of Portugal, both at the age of sixteen. After the first year of marriage without offspring, the court physicians used some curative methods, such as applying bloodletting to the legs, in order to improve the queen’s fertility, a situation that weakened her health. Finally, Maria Manuela of Portugal had her first pregnancy in September 1544 and her first delivery on 8 July 1545 (the birth was long and difficult due to an anomalous presentation). The birth was attended by the family doctor, Luso (known to be a dwarf with a very large head), and Villalobos, the royal family doctor, as well as two midwives. As a result of the great obstetric manipulation, she died of a puerperal infection on 12 July 1545, after twenty months of marriage and at the age of eighteen [32,33].

The monarch, now twenty-six years old, remarried Mary Tudor, Queen of England, aged thirty-eight. The marriage was arranged by Charles I, Philip II’s father, making it a marriage of convenience. The age and geographical distance between the two did not encourage offspring. In 1555, the queen had a fictitious pregnancy and childbirth, which caused Philip II to accompany the queen during most of her pregnancy and the beginning of the birth. Two years later (1557), this queen had her second fictitious pregnancy, but on this occasion she faced the situation without her husband’s company, due to the embarrassment it meant for the monarch. Finally, Mary of Tudor died a year later due to a flu epidemic [34,35].

Philip II’s third marriage took place when he was thirty-three years old and his new wife, Isabella of Valois, was fourteen. She was known as “Isabella of Peace”, for with her came harmony between Spain and France through the Treaty of Cateau Cambresis (1559). The marriage was celebrated without the queen’s menarche, a situation that prompted her mother (Catherine de Medici) to send remedies from France to cause the arrival of her first menstruation [19]. The latter also arranged for doctors and even an apothecary to accompany her to Spain in anticipation of her pregnancies [36]. In fact, mother and daughter maintained a fluid communication by means of letters, where they discussed issues related to sexuality and natural remedies to favour offspring, among others. Finally, her first menarche appeared in 1560, at the age of fifteen [37].

Her first pregnancy, four years later, ended in miscarriage. It can be said that her health was preserved by different socio-health institutions. Her sexual and reproductive problems were treated by the doctors Montguyon and Burguensis, sent by her mother from France. She was also treated by the doctors of the royal family, Juan de Santiago and Antonio de Paz y de la Vega, and by the surgeon Juan Fragoso [19]. She even received spiritual help from the Spanish subjects through prayers, fasting, processions and the transfer of the image of Our Lady of Atocha to the Alcázar, as well as the administration of the Holy Unction.

From the second pregnancy of this queen, the infant Isabel Clara Eugenia was born in 1565. Before the birth, she made a will in which it was written that she would give Juan de Santiago and Antonio de Paz 500 ducats, and the doctor de la Vega 300 ducats. Montguyon, her personal physician, was to receive 1000 ducats. Finally, six years after her first period (12 August 1566) Isabel Clara Eugenia was born with the participation of the doctors Vega y Olivares, Andrés Vesalio, Chacón and Mena. A year later, she had her third pregnancy, from which Catalina Micaela was born (1567), being cared for by the comadre Mª Álvarez de Porras, to whom 2200 reales were given. Her fourth and last pregnancy was in 1568, and she suffered a miscarriage at five months. Isabella of Valois died on 3 October 1568, three months after the infant Charles [3,37].

**Figure 2:**
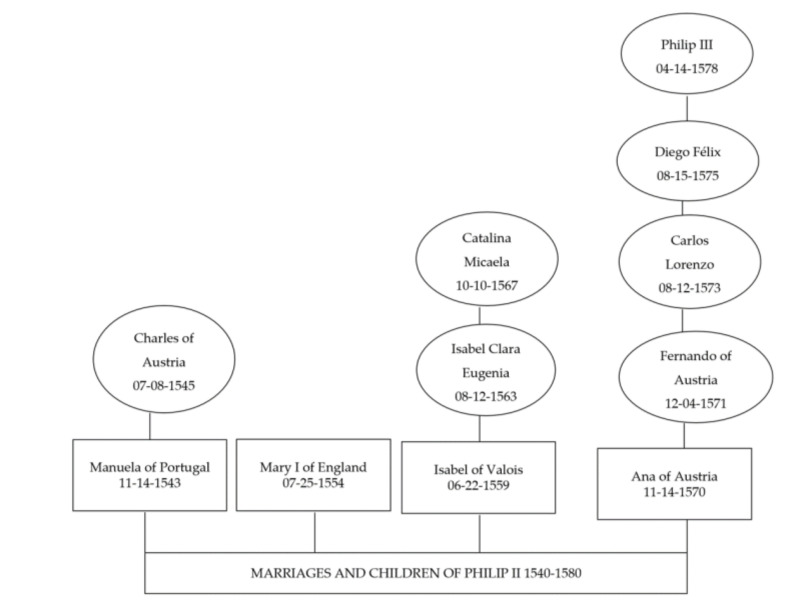
Women and children of Philip II (1540–1580). Source: authors’ own elaboration.

Finally, Philip II, now aged forty-two, married his niece Anne of Austria, who was twenty-one years old. This marriage, which took place in 1570, produced four sons and a daughter. The first was Prince Ferdinand, who died at the age of seven. The second son, Carlos Lorenzo, was born on 12 August 1573 and died at the age of two. His third son, Don Diego Felix, was born on 12 July 1575 but died of smallpox on 21 November 1582. Finally, her fourth son was born in 1578, Philip III, who eventually became King of Spain. Anne of Austria’s fifth child was born on 14 February 1580, named Maria, and died on 14 August 1583. Finally, Anne of Austria died on 26 October 1580, while pregnant with her sixth child. This situation again indicates the primary function of these women and queens within the court: the perpetuation of the dynasty through reproduction [38] (Figure 2).

### 3.2. Wet Nurses and Babies and Bonding through Milk: The Value of Work

This section describes and emphasises the figure of the wet nurse, specifically in the Spanish royalty, and her relationship with the infants, highlighting both the benefits and the retribution they acquired for their work. It is worth noting that at this time the wet nurses of the royal court generally received the distinction of “Doña”, acquiring payment in cash and household goods for their work. This was in addition to some benefits, such as jobs for their husbands, sons or relatives in subordinate occupations in the royal household. The first wet nurse to appear in the palace is related to the birth of the first child of King Philip II and Maria Manuela of Portugal, who was born with signs of abnormality. This queen died of a puerperal infection four days after giving birth (12 July 1545) [17,39].

The newborn, Charles II, was breastfed for 15 months by a wet nurse. His only known wet nurse was Dª Ana de Luzón, wife of D. Gaspar de Osorno, noble descendants of Tudela. This wet nurse was dismissed due to lactation problems. Nevertheless, Philip II ordered his father Charles I to offer this family a good dowry in order to provide this wet nurse with a more comfortable life [17].

To breastfeed Isabella Clara Eugenia (1565), the first child of his third marriage, the royal family physicians sought fifty wet nurses from noble families, who had to be free of all Moorish or Jewish blood. The doctors of the Chamber chose only three wet nurses, dismissing the rest. In fact, Isabella Clara Eugenia suckled from four wet nurses. The origin of only the first is known: Ana López, a resident of Ávila, from a village near Balsaín, who was soon dismissed. She breastfed the Infanta Isabel on the day she was born and two days later, for which this woman was given two hundred ducats of aid [17,40].

This first wet nurse was replaced by Doña Beatriz de Mendoza, who nursed Isabel Clara for four months. However, this infant was on the point of starving to death, probably due to the shortage of milk from her first wet nurse (Dª Ana López). The payment that Doña Beatriz received amounted to two hundred ducats and thirty thousand maravedís (mrs) as an annual pension, in addition to an office for her husband [41].

The third wet nurse, Doña María de Oviedo, nursed the infant for almost twelve months and was considered to be the best wet nurse. For this reason, she performed the functions of a wet nurse and was granted sixty thousand maravedís for life, issued in the tercias of Ciudad Rodrigo (Salamanca), which is why it is suspected that this was the place of her birth and may even have been her place of retirement. Finally, for five months Doña María de Rivas nursed the infant, who began her office after Corpus Christi. She was paid two hundred ducats and was also allotted thirty thousand maravedís as a lifetime salary [17,42].

It was rare for a single wet nurse to continue the entire upbringing until the infants were weaned; this only happened with the Infanta Catherine Micaela, second daughter of Philip II and Isabella of Valois. She was nursed for twenty-two months by Doña María de Messa, who received one hundred thousand maravedís for life, as well as the bed she used and the cradle for the infant. She also secured her daughter a job as a valet [33,39].

From the fourth marriage of Philip II and Anne of Austria was born Prince Ferdinand, who was suckled by eight wet nurses. The first of these was Doña María de Terán, who breastfed him for twenty-four days, from the day he was born (4 December 1571) until the night of Saint John the Evangelist. Ferdinand had two of the aforementioned wet nurses, Doña María de Mesa, who breastfed him for eight months, and Doña María de Rivas, who returned to the Palace to nurse the prince for a month. The prince was breastfed for almost three years by five other wet nurses, who were named Doña Luisa Fernández and Isabel Grado Mayor, the latter of whom served twice in the Palace and was a resident of Casarrubios (a village in the province of Toledo). At first, she nursed for more than nine months, and then, when “The Relative of Accountant Santa Cruz, who came from Toledo” was only able to feed the Prince for four days, she returned for another twenty-one days. She was succeeded by the wet nurse Juana Bautistina, who lived in Madrid. Leonor de Garay also breastfed until Prince Ferdinand was weaned [17,33,39].

In addition, there were many women brought in for selection, but they were rejected by the doctors of the Chamber. In total there were eleven, of whom the provenance of almost all are known. Despite not having performed the function of wet nurses, these women were given an amount ranging from three hundred ducats for the most favoured to fifty for the least rewarded [17] (Table 2).

On 12 August 1573, the feast of Saint Clare, Carlos Lorenzo was born and was subsequently raised by two wet nurses. Doña María de Neira, wife of Juan García, a neighbour of Segovia, nursed the infant for two months [43]. Then, Doña Isabel Galindo, wife of Francisco de Salablanca, was wet nurse for a year and a half, until 11 April 1575; she received eighty thousand maravedís plus the bed of damask and blue velvet in which she slept and the infant’s bed, for having weaned him. Carlos Lorenzo died on 9 September 1575 [44].

On 12 July 1575, Don Diego Félix was born, and he suckled from five women. The first was María de Valdés, a resident of Las Navas, from whom he suckled for only one month. Doña Leonor de Garay, the wet nurse who two years earlier had weaned Prince Ferdinand, stayed with the infant “the night of 12 August 1576 when he came to the palace and for three more days”. Doña Felipa del Mármol, daughter of the secretary Mármol and wife of the licentiate Montemayor, was given a lifetime pension of sixty thousand maravedís a year for the eight months she remained in service, and the next wet nurse, Doña Magdalena Pachón, married to a silversmith, who nursed Don Diego for four months, is known to have received clothes, such as a sackcloth and a doublet of brown satin, as well as three hundred ducats. The last wet nurse was Isabel Páez de la Fuente, wife of Francisco de Espina, who was also his nursemaid; she arrived at the palace on 1 August 1577 [17]. She gave milk until he was weaned, for a total of three months, after which she remained in service at the palace. She was assigned 50,000 maravedís for life. Diego Felix died of smallpox on 21 November 1582 [17,19,33] (Table 3).

Philip III, who eventually became King of Spain, was born in 1578, although the date is uncertain, being either 13 or 14 April. He was nursed by Doña Leonor de Garay, who had weaned Prince Don Fernando (1574) and had nursed Infante Don Diego for a short time (1576). Five wet nurses fed the infant for a short time. Doña Francisca de Urbina was a resident of Barajas. Francisca de Torquemada was wet nurse for ninety-one days and then “also served in the açafate of his highness due to the illness of Doña Phelipa de Espinosa” for a little over two months [45]. On 27 October 1579, when he was just over a year- and-a-half old, he was weaned by Doña Mariana de Vargas, who was his first wet nurse, and this time he nursed four months. She was given “a bed of crimson damask in which she slept” and a pension of fifty thousand maravedís a year [46] (Table 4).

### 3.3. Application of Empirical Medicine: Health Promotion and Care

The following are the natural remedies, according to the knowledge and beliefs of the time, related to the care for improving and strengthening women’s health in the period studied (1540–1580). Due to the queens’ need for offspring and the high maternal and foetal mortality rate at the time, as well as the limited development of medicine, a series of remedies based on empirical practice were applied.

Special attention should be paid to the problems and illnesses specific to women in the period under study. A series of prescriptions, ointments and plasters related to menstruation, pregnancy, childbirth, child rearing, breasts, milk and milk withdrawal were applied to women [47]. This is the origin of the importance of the figure of the wet nurse; although it is known that this profession has been regulated in Spain since the 12th century, it was during the 16th century when her presence was greater at court [48].

During childbirth, natural remedies were used and women were attended by midwives and court physicians [49]. Such is the case of the birth for Manuela of Portugal, the first wife of Philip II, where the Eagle stone (limonite or iron oxide) was used, whose purpose was to produce vasodilation of the perineal veins to aid in childbirth [19].

Another type of practice performed on these women was bloodletting, a remedy carried out by Diego Ortega (a barber from Toledo) on Isabella de Valois, the third wife of Philip II, when she contracted smallpox. This queen was also treated by Gaulish doctors, who used remedies such as salt water and pigeon’s blood with cream, applied to the eyes, to prevent the after-effects of the disease, especially on the face. However, it should be noted that these practices were carried out against the scientific evidence of the time; as early as 1541, Damian Carbon wrote the “Book of the art of midwives or godmothers”, in which he indicated the prohibition of bloodletting, as well as restricting purgatives and enemas [51].

It is known from recipe books that an ointment was used to remove milk, which was applied four or five hours after childbirth. In fact, a cloth soaked in pink water was placed on each breast, the breasts were banded, and these cloths were changed once a day. In addition, to avoid contractions after childbirth, orange blossom water with goat’s cloth was put on the belly, which was changed at each meal, and was used for three days. In addition, a hen was boiled with parsley and saffron, and the mother was given the concoction to drink while it was hot [52].

Empirical medicine was therefore oriented around recipes related to health, beauty and feminine hygiene, and the use of aphrodisiac remedies is even known. One of the requirements that wet nurses had to fulfil was related to their beauty and even their hygiene [49].

It is worth noting that some women were rejected for the job of wet nurse because they did not meet the standards of beauty, sometimes due to dental problems. Thus, for facial skin care, a remedy was prepared from oil, honey, wine and vinegar. There were remedies to reduce odours, based on water, pills, oils, flower baths, roses, violets, orange blossom and amber [47].

The most relevant health problems were those related to the throat, mouth, stomach, liver, lungs, heart, bones and joints. One remedy used for asthma was to stew eggs with cat’s ointment. Regarding the circulatory system, there was a remedy for blood flow, which was prepared with frankincense, nutmeg and cloves, among other ingredients. All of these were mixed together and given to the sick person to drink for nine mornings in order to heal them [18,53].

It is remarkable the amount of remedies used to treat “madness”, an illness that sometimes occurred due to consanguine marriages carried out between cousins, aunts, uncles and other relatives. These remedies could be ingested or used in the form of ointments and herbal remedies. In some cases, superstition was involved [52].

## 4. Discussion

Historically, and for centuries, human nutrition has been the only way to guarantee the health and survival of the newborn, and its failure has been one of the most important causes of death. While it is true that the research maintains that breastfeeding by wet nurses favoured the survival of royal infants, this was not the case for newborns raised by wet nurses in institutions or foundling homes, and even breastfeeding did not guarantee the survival of royal infants in all cases, as some inevitably died of infectious and contagious diseases. Therefore, it can be stated that breastfeeding, together with hygienic and sanitary conditions and access to medical resources made it possible for the royal infants breastfed by wet nurses to survive longer than those raised in lower social strata. Neonates nursed by non-royal wet nurses generally did not have the same sanitary conditions and had fewer health resources. For this reason, breastfeeding was not sufficient in these cases. Thus, the office of wet nurse was undoubtedly a guarantee of health in royalty, but it did not ensure the survival of infants, just as breastfeeding, despite its nutritional and immune components, did not guarantee the survival of children from lower social classes in that period. The subject of newborn feeding has been present in the medical treatises of great thinkers, philosophers, physicians and historians throughout the centuries. In the vast majority of cases, the approach to the subject was centred on the figure of the mother and the wet nurse, valued from the point of view of moral or religious indoctrination, considering the woman as an ignorant, hysterical or capricious being [4,5].

The great importance given to the reasons and advice on breastfeeding that sages and scholars, since the time of Aristotle, have used to justify its convenience for both mother and child is surely to be found in the fact that many women who could afford it were unwilling or unable to breastfeed [53]. This situation highlights the weak balance between the natural, the cultural and the social. Since the beginning of society, a difference has been established between women who breastfed their children and those who, for cultural or social class reasons, did not engage in this practice [4]. In fact, different tests are described in the work of Oribasius of Pergamon, 4th century AD, to check the quality of the milk of wet nurses and whether they were fit to care for and feed a child belonging to the nobility [8].

Works on wet nurses have been very prolific in records, legends and even manuscripts. Thus, in the works of great physicians of antiquity such as Soranus of Ephesus, Oribasius, Mesitheus, Etius and Galen, advice to be taken into account for wet nursemaids was extracted and was followed well into the 20th century [7]. In fact, Abou Aly [8] shows us, through the texts of Ancient Greek physicians, the recommendations to be taken into account when choosing a good wet nurse. In this sense, Knibiehler [56] made an important contribution with his work on the non-maids of the wealthiest classes in France during the Ancien Régime, becoming one of the basic references on the practices related to the figure of the wet nurse.

Such is the importance of this profession carried out by women that authors such as Colmenar have shown us the legislation and regulations relating to wet nurses as a measure aimed at protecting infants and the children of the upper classes, but above all to protect the heirs of noble families [57].

However, what can be said about the role of breastfeeding in noble families, and specifically in the royal family of 16th century Spain, one of the most powerful and important royal families in history? Raising a child in the 16th century was not an easy task; infant mortality was very high, and to survive beyond the age of two or three was a real challenge. Neither princes nor kings escaped this sad reality, with Philip II standing out in this respect; he had eight legitimate children with four wives, of whom only three survived [17,18]. This situation highlights the importance of the wet nurse for the suckling of infants, as these women could help royalty by increasing infant survival, by excluding queens and birth mothers from the suckling process, and by increasing the frequency of childbearing to expand the power of the ruling classes.

Faced with this terrible situation and given the importance of the royal heirs for the very survival of the monarchy, it was logical that care and attention should be paid to the young infants and their royal mothers. One of the aspects of greatest consideration was the choice of suitable and sufficient breeding mistresses, given the high mortality rate among infants, as has already been studied in this work. It was also necessary to guarantee sufficient food for the infant royal children. In this regard, Cabrera [32] provides a historical overview of the wet-nursing profession, including the auxiliary nursing carried out by the most modest women, as well as the wet nurses employed in the royal households, some of whom were immortalised by court painters together with the infants in their care. On the other hand, Del Hoyo [58] studied the qualities required of an upper-class wet nurse and the physical and anatomical examination she had to undergo.

In the court of Philip II, wet nurses and housekeepers enjoyed a good reputation, and their social standing was so high that many were called “Doñas” and were given exquisite gifts. The most productive of them obtained certificates of nobility for their husbands and large lifetime salaries.

However, the court of Philip II established health criteria, as these women had to be healthy, beautiful and with milk—if possible, a lot of milk. Ethnic-religious prohibitions were also created, as wet nurses had to be old Christians [59] and, in many cases, with a certificate of cleanliness of blood, which meant that they could not be Jewish or Muslim, or even descendants. Finally, there were the moral considerations, as they had to have good customs, which had to be justified directly by the church [60].

Such was the importance of this figure at the court of Philip II that a letter sent from Madrid on 5 December 1588 by King Philip II to one of his daughters, Catherine Micaela, Duchess of Savoy, was preserved, in which the monarch was interested in the health of his three grandchildren and mentioned the mistresses of the brood: “[…] and also that my grandchildren are so well and that what Vittorio Amadeo had is past, and it is very good to give them the mistresses when necessary” [61].

The presence of wet nurses was essential, as this work brought great benefits to these women and their families. In this sense, Borrel examines the reasons that could coexist with the economic contribution for a woman to decide to work as a wet nurse in Girona during the 17th and 18th centuries [62].

In addition, the court encouraged queens to devote themselves to their own bodily care and to exercise other functions and obligations within the court, apart from the search for an heir. This situation led them to use the empirical medicine of the time through health, beauty and curative methods to improve their image and to avoid the epidemics of the time (influenza, smallpox) and even to remove breast milk and control bodily alterations after childbirth. The queens had to look like healthy and attractive women to a man who wanted to perpetuate his dynasty on the throne through his children [63]. Therefore, the figure of the wet nurse is important because she substituted for the natural mother in breastfeeding, in the supervision of the child’s care and in education so that she could carry out the obligations of the court and of society, allowing queens to continue having children and thus perpetuate the lineage and the family name in power for generations.

It is important to consider that, by avoiding breastfeeding, the mother restarted the reproductive cycle as soon as possible, which favoured an increase in the number of births [64]; despite the fact that the infants had good wet nurses and the best nurses of the time, mortality in early infancy was very high [17,18]. In fact, the court of Philip II was not the only one that enjoyed the presence of milkmaids, as it is known that in the Cortes of the Castilian kings they were common, starting at least in the 12th century, even regulating the criteria for the selection of wet nurses in the Castilian constitutional body, in the very same Partidas. Thus, in the second partida, in the third law of chapter VII, the characteristics that these wet nurses had to have were set forth [65].

## 5. Conclusions

The presence of wet nurses was essential, as this work brought great benefits to these women and their families. The selected wet nurses were the driving force in promoting the health of the infants through the breastfeeding process. This situation was motivated by the hereditary systems of the prevailing monarchy, through the legal regulation of marriage that governed the queen’s functions within the court. This was due to the private life of the monarchy, the relationship between sexuality and reproduction, and the formal institutional codes of marriage as a means of ensuring offspring.

At a crucial moment in 16th century Spain, the woman as queen, mother and wife, together with the invaluable help of the wet nurses acted as a device of monarchy, a tool of loyalty, example, cohesion and subjection in the face of bad times, in an empire, the Spanish empire, where, according to legend, the sun never set.

## Figures and Tables

**Table 1:** Thematic blocks related to the references.

Database	Search Strategy	Limits	Points Extracted	Reference
PubMed	Special care OR culture care AND infant AND health promotion OR breastfeeding AND historical research OR wet nurse AND care AND maternal AND society AND infant mortality OR child survival	Title Article English/ Spanish/ Portuguese/Arabic	Woman and queen: fertility Doctors Queens: epidemic diseases Pseudogestation, royal midwives Sexuality and natural remedies Lady wet nurses Family privileges Adjoining localities Payments: household goods and mrv Lactation period Health care: empirical medicineBeauty and feminine care administered by midwives	[32,33] [34,35,36] [3,37,38] [39,40] [41,42,43,44,45,46,47,48,49,50,51,52,53]
Science Direct				
CINHAL				
Scopus				
Cuiden				
Google Scholar				

Source: authors’ own elaboration.

**Table 2:** Origin of wet nurses rejected by doctors of the royal family.

Wet Nurse	Town of Origin	City
two	Arévalo	Avila
one	Del Bisso (Viso de San Juan)	Toledo
one	Alcalá	Madrid
one	Villanueva de los Infantes	C. Real
one	Torrejón de Ardoz	Madrid
Aldonza de Valdes	Wife of Fernando Ramirez, secretary of the Inquisition	Murcia
two	Illescas	Toledo
one	Valdemoro	Madrid
one	-	Toledo

Source: authors’ own elaboration.

**Table 3:** Wet nurses who breastfed different infants born to Philip II.

Wet Nurse	Queens: Mothers of Infants	Infants	Lactation Time
Dª María de Rivas	Isabel de Valois (3rd woman)	Isabel Clara Eugenia (2nd daughter Philip II)	Five months
	Ana de Austria (4th woman)	Fernando de Austria (4th child Philip II)	Eight months
Dª María de Messa	Isabel de Valois (3rd woman)	Catalina Micaela (3rd daughter Philip II)	Twenty-two months
	Ana de Austria (4th woman)	Fernando de Austria (4th child Philip II)	One month
Dª Leonor de Garay	Ana de Austria (4thwoman)	Fernando de Austria (4th child Philip II)	Weaned the infant in 1574
		Diego Félix (6th child Philip II)	3 Days
		Felipe II (7th child Philip II)	Not known

Source: authors’ own elaboration.

**Table 4:** Queens/wives of Philip II and their children as well as wet nurses and their renumeration.

Queens: Wives of Philip II	Children	Wet Nurse	Remuneration	Duration of Breastfeeding
Manuela de Portugal (1st Wife)	Carlos de Austria	Dª Ana de Luzón	Unknown dowry	Fifteen months
María I de Inglaterra (2nd Wife)	No offspring (2 False Pregnancies)	-	-	-
Isabel de Valois (3rd Wife)	Isabel Clara Eugenia	Dª Ana López	Two hundred ducats	Three days
		Doña Beatriz de Mendoza	Two hundred ducats + thirty thousand mrs annual pension + husband’s office	Four months
		Doña María de Oviedo	Sixty thousand mrs annual pension	Fourteen months
		Doña María de Rivas	Two hundred ducats annual pension + thirty thousand mrs	Five months
	Catalina Micaela	Doña María de Messa	One hundred thousand mrs annual pension + beds + job for his daughter	Twenty-two months
Ana de Austria (4th Wife)	Fernando de Austria	Doña María de Terán	Two hundred ducats + one hundred escudos in gold + dress of 32,509 mrs.	Twenty-four days
		Doña María de Mesa	Unknown dowry	Eight months
		Doña María de Rivas	Black taffeta clothingof 21,232 mrs	One month
		Luisa Fernández	Four shirts and two headdresses	One month
		Isabel Grado Mayor	Two hundred ducats	Nine months + twenty-one days
		Juana Baustitina	One hundred thousand for life + dress of 9400 mrs.	-
		Doña Leonor de Garay	Not known	She weaned him at the age of three (1574)
	Carlos Lorenzo	Doña María de Neira	Not known	Two months
		Doña Isabel de Galindo	Eighty thousand mrs for life + beds	One year and a half
	Diego Félix	María de Valdés	Not known	One month
		Doña Leonor de Garay	Not known	Four days
		Doña Felipa de Mármol	Life pension of 60,000 mrs per annum	Eight months
		Doña Magdalena Pachón	Clothes and three hundred ducats	Four months
		Isabel Páez de la Fuente	50,000 mrs for life	Three months
	Felipe III	Doña Leonor de Garay	Not known	Not known
		Doña Francisca de Urbina	Not known	Not known
		Doña Felipa de Espinosa	Not known	Not known
		Francisca de Torquemada	Not known	Ninety-one days
		Doña Mariana de Vargas	Crimson damask bed + fifty thousand mrs per annum	Four months

Source: authors’ own elaboration.
